# Semaglutide May Ameliorate Fibrosis and Inhibit Epithelial–Mesenchymal Transition in Intrauterine Adhesion Models

**DOI:** 10.3390/ijms25116196

**Published:** 2024-06-04

**Authors:** Luming Wu, Yue Zhan, Yiqing Wang

**Affiliations:** 1 State Key Laboratory of Bioactive Substance and Function of Natural Medicines, Institute of Materia Medica, Chinese Academy of Medical Sciences & Peking Union Medical College, Beijing 100050, China; ludomwu@163.com; 2 Key Laboratory of Preclinical Study for New Drugs of Gansu Province, School of Basic Medical Sciences & Research Unit of Peptide Science, Chinese Academy of Medical Science, 2019RU066, Lanzhou University, Lanzhou 730000, China; zhany20@lzu.edu.cn

**Keywords:** Semaglutide, intrauterine adhesions, fibrosis, epithelial–mesenchymal transition

## Abstract

The purpose of this study was to explore the effect of Semaglutide on intrauterine adhesions and discover new drugs for such adhesions. In this study, the cell model was simulated by TGF-β1-induced human endometrial epithelial cells, and the animal model was established through mechanical curettage and inflammatory stimulation. After co-culturing with TGF-β1 with or without different concentrations of Semaglutide for 48 h, cells were collected for RT-qPCR and Western blotting analyses. Three doses were subcutaneously injected into experimental mice once a day for two weeks, while the control group received sterile ddH2O. The serum and uterine tissues of the mice were collected. HE and Masson staining were used for the uterine histomorphological and pathological analyses. RT-qPCR and Western blotting were used for mRNA and protein expression analyses. Serum indicators were detected using ELISA kits. The results showed that Semaglutide significantly reduced the mRNA levels of fibrosis indicators ACTA2, COL1A1, and FN and inflammatory indicators TNF-α, IL-6, and NF-κB in the two models. Semaglutide improved endometrium morphology, increased the number of endometrial glands, and reduced collagen deposition in IUA mice. The results also showed that Semaglutide could inhibit vimentin, E-Cadherin, and N-Cadherin in the two models. In summary, Semaglutide can ameliorate fibrosis and inflammation of intrauterine adhesions as well as inhibit epithelial–mesenchymal transition in IUA models.

## 1. Introduction 

Intrauterine adhesion (IUA) is one of many diseases that affect female reproductive health, with clinical manifestations such as irregular menstruation, cyclic pelvic pain, and even infertility and recurrent miscarriage. Studies have shown that the incidence of IUA among women of childbearing age is as high as 31.6% [1,2]. Currently, hysteroscopy is considered the gold standard for the clinical diagnosis of IUA and is also the only recognized first-choice treatment. In addition, an IUD is placed in the uterus to physically separate the uterus from re-adhesion, and estrogen is supplemented to stimulate endometrial regeneration [3]. However, surgery has the disadvantages of poor patient compliance, a high recurrence rate, and many complications [4]. IUDs such as balloons and stents can physically separate the inner walls of the uterus but cannot repair the damaged endometrium. IUDs and surgical procedures may also cause secondary damage to the uterus, increase irritation to the uterus, and increase the risk of inflammation [5,6,7]. Estrogen can promote endometrial growth, but there is currently no unified dosage or administration time, and its clinical application is still controversial [8,9]. Therefore, it is of great significance to find new, effective drugs for the treatment of IUA.

Semaglutide is a novel glucagon-like peptide-1 (GLP-1, a 29-amino-acid-residue incretin hormone) receptor agonist with 94% homology, approved by the Food and Drug Administration for the treatment of diabetes in December 2017. Research on Semaglutide is currently very active. It has been found that Semaglutide has many benefits for the human body, including cardiovascular protection and weight loss, and its effect is superior to other GLP-1RAs such as liraglutide [10]. Studies have shown that GLP-1 can exert anti-inflammatory, antioxidant, and anti-fibrotic effects through glucose-dependent and glucose-independent mechanisms. Multiple studies have shown that GLP-1 receptor agonists, including natural GLP-1, exenatide, and liraglutide, can reduce monocyte vascular adhesion by reducing monocyte adhesion and macrophage aggregation, thereby improving cardiac function and reducing atherosclerosis in the blood vessels of atherosclerotic mice [11,12]. Studies in renal fibrosis have shown that liraglutide inhibited the activation of downstream classical and non-canonical signaling pathways (p-Smad3 and p-ERK1/2) by reducing the expression of TGF-β1 and its receptors, thereby inhibiting TGF-β1-induced epithelial–mesenchymal transition (EMT) in NRK-52E cells, which effectively prevented the fibrosis process in chronic kidney disease [13]. In fact, there have been studies exploring the use of GLP-1 receptor agonists alone or in combination with other drugs to improve the degree of fibrosis in the liver, lung, and other body parts [14,15,16].

Compared with other GLP-1 receptor agonists, there are relatively few studies on Semaglutide in the treatment of fibrotic diseases or intrauterine adhesions. Therefore, this experiment explores the effect and potential mechanism of Semaglutide on IUA cells and mouse models in order to discover new drugs for the treatment of intrauterine adhesions and provide new ideas for the treatment of other fibrotic diseases.

## 2. Results

### 2.1. Effect of Semaglutide on Cell Viability

**Figure 1:**
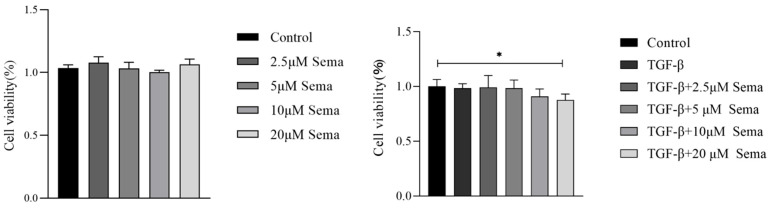
Viability assay of Semaglutide on HEECs induced with or without TGF-β. * *p* < 0.05.

As shown in Figure 1, we examined the effect of Semaglutide on the viability of human endometrial epithelial cells (HEECs) induced with or without TGF-β. We found that at a concentration of 20 μM, Semaglutide had no effect on the viability of HEEC without TGF-β but had an effect on the viability of HEEC induced by TGF-β (*p* < 0.05). Based on this result, we set three concentrations of Semaglutide (0.1 μM, 1 μM, and 10 μM) for subsequent cell experiments. 

### 2.2. Semaglutide Downregulated the Expression of Fibrotic and Inflammatory Factors in TGF-β1-Induced HEECs

**Figure 2:**
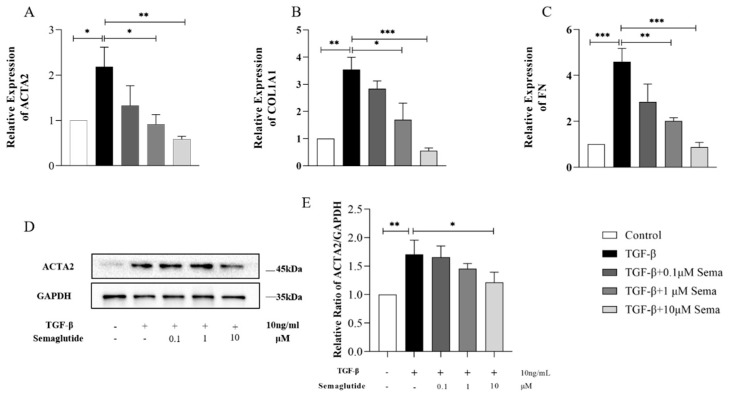
RT-qPCR analysis of the relative mRNA expression levels of ACTA2 (**A**), COL1A1 (**B**), and FN (**C**) in TGF-β-induced HEECs treated with or without Semaglutide. Western blot analysis of the protein expression level of ACTA2 (**D**,**E**) in TGF-β-induced HEECs treated with or without Semaglutide. Data are expressed as mean ± SD. * *p* < 0.05, ** *p* < 0.01, and *** *p* < 0.001.

As shown in Figure 2, after induction by TGF-β, the mRNA expression of ACTA2, COL1A1, and FN in HEECs increased significantly (*p* < 0.05). After intervention with Semaglutide, the expression of these indicators decreased. We also found that this down-regulatory effect of Semaglutide was concentration-dependent. At the same time, we detected the protein expression level of ACTA2 in HEECs via Western blotting. As shown in Figure 2D,E, compared with the control group, TGF-β significantly increased the expression of ACTA2 (*p* < 0.01). As the concentration of Semaglutide increases, the expression of the ACTA2 protein gradually decreases, which is consistent with the results of mRNA expression. 

**Figure 3:**
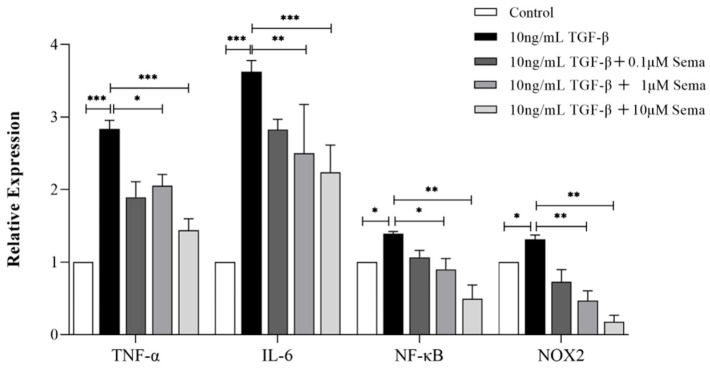
RT-qPCR analysis of the relative mRNA expression levels of TNF-α, IL-6, NF-κB, and NOX2 in TGF-β-induced HEECs treated with or without Semaglutide. Data are expressed as mean ± SD. * *p* < 0.05, ** *p* < 0.01, and *** *p* < 0.001.

As shown in Figure 3, we detected that the mRNA expression of key factors related to inflammation and oxidative stress, TNF-α, IL-6, NF-κB, and NOX2, was significantly increased after TGF-β induction (*p* < 0.05) and significantly decreased after Semaglutide intervention (*p* < 0.01).

### 2.3. Semaglutide Improved Endometrial Morphology and Downregulated the Expression of Fibrotic and Inflammatory Factors in IUA Mice

**Figure 4:**
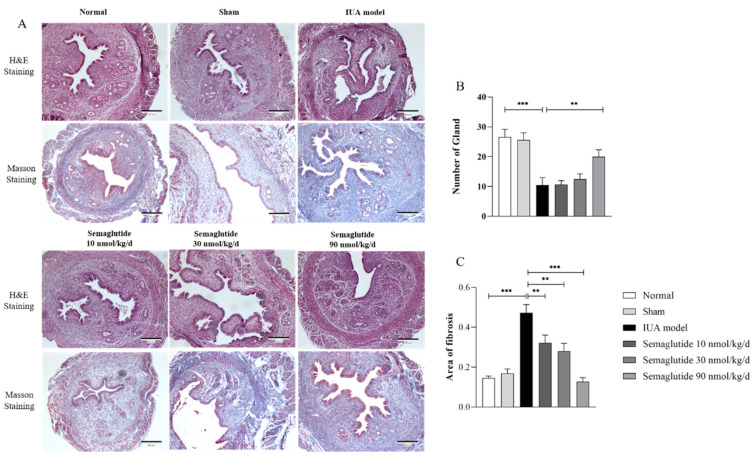
Histological structures of the uterus in the six experimental groups (H&E and Masson staining). (**A**) Analysis of the number of glands (**B**) and fibrotic area (**C**) in the endometrium in each group at 14 days after IUA model establishment. The scale bar in the figure is 200 μΜ. Data are expressed as mean ± SD. ** *p* < 0.01 and *** *p* < 0.001.

As shown in Figure 4, compared with the normal group, there was no significant difference in the degree of endometrial fibrosis in the sham group. The number of glands and blood vessels in the uteri of mice in the model group, low-dose group, and middle-dose group was significantly reduced (*p* < 0.01), while these numbers increased in the high-dose groups after treatment (*p* < 0.05). Compared with the normal group, collagen deposition in the uteri of mice in the model group increased significantly (*p* < 0.0001). After treatment, collagen fibers in each treatment group were reduced by varying degrees, and the difference was statistically significant (*p* < 0.01).

**Figure 5:**
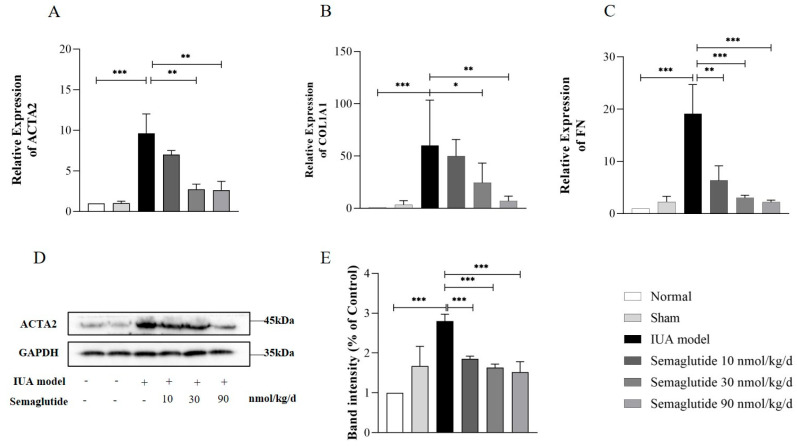
RT-qPCR analysis of the relative mRNA expression levels of ACTA2 (**A**), COL1A1 (**B**), and FN (**C**) in the uteri of mice in each group. Western blot analysis of the protein expression level of ACTA2 in the uteri of mice in each group (**D**,**E**). Data are expressed as mean ± SD. * *p* < 0.05, ** *p* < 0.01, and *** *p* < 0.001.

Consistent with the cellular results, similar changes in fibrotic markers were detected in the uterine tissues of IUA mice. The mRNA expression levels of ACTA2, FN, and COl1A1 were significantly increased (*p* < 0.001), while Semaglutide significantly downregulated these changes (*p* < 0.05). (Figure 5A–C) The protein expression level of ACTA2 also significantly increased in the IUA model (*p* < 0.001) and decreased after treatment with Semaglutide (*p* < 0.001) (Figure 5D,E).

**Figure 6:**
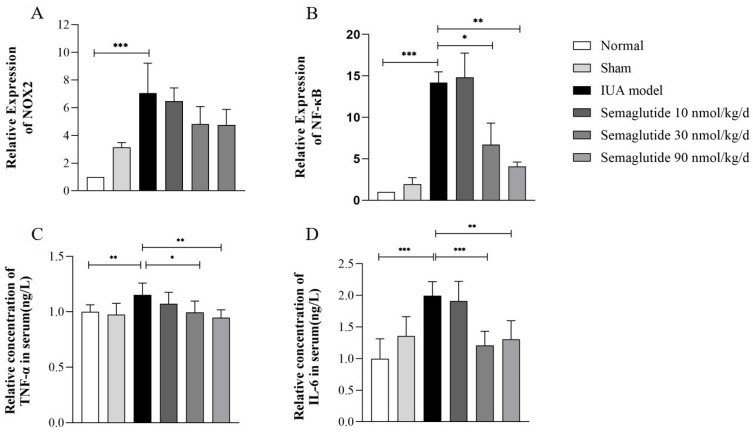
RT-qPCR analysis of the relative mRNA expression levels of NOX2 (**A**) and NF-κB (**B**) in the uteri of mice in each group. ELISA analysis of the serum levels of TNF-α (**C**) and IL-6 (**D**) in mice in each group. Data are expressed as mean ± SD. * *p* < 0.05, ** *p* < 0.01, and *** *p* < 0.001.

As shown in Figure 6, after treatment, the mRNA expression of NF-κB and the serum levels of TNF-α and IL-6 were significantly reduced (*p* < 0.01), but a reduction in the mRNA expression of NOX2 in uterine tissue was not detected. 

### 2.4. Semaglutide May Ameliorate Fibrosis by Inhibiting Epithelial–Mesenchymal Transition

**Figure 7:**
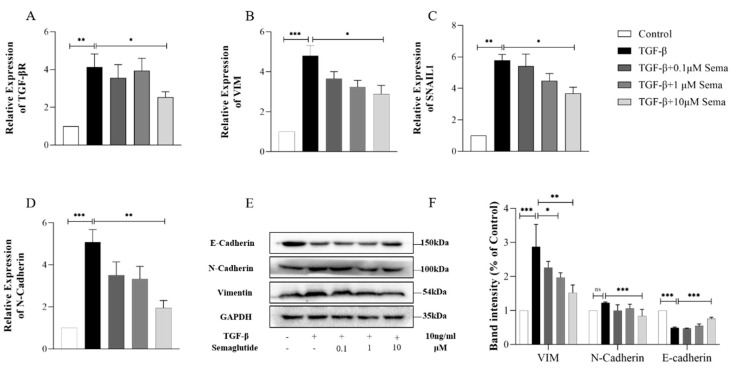
RT-qPCR analysis of the relative mRNA expression levels of TGF-βR (**A**), vimentin (**B**), SNAIL1 (**C**), and N-Cadherin (**D**) in the TGF-β-induced HEECs in each group. Western blot analysis of the protein expression level of E-Cadherin, N-Cadherin, and vimentin in the TGF-β-induced HEECs in each group (**E**,**F**). Data are expressed as mean ± SD. * *p* < 0.05, ** *p* < 0.01, and *** *p* < 0.001.

Compared with the control group, the mRNA expression levels of TGF-βR, SNAIL1, VIM, and N-Cadherin in TGF-β-induced HEECs increased significantly (*p* < 0.05). Except for TGF-βR, the expression levels of the other three indicators were reduced after the intervention of Semaglutide, and there was a significant difference at the highest concentration of 90 nmol/kg/d (*p* < 0.05) (Figure 7A–D). Western Blot showed that with Semaglutide intervention, the protein expression of VIM (*p* = 0.0048) and N-Cad (*p* = 0.0476) were significantly reduced, and E-Cad increased (*p* = 0.0221) in TGF-β-induced HEECs (Figure 7E,F), further suggesting that Semaglutide has an inhibitory effect on EMT in TGF-β-induced HEECs. 

**Figure 8:**
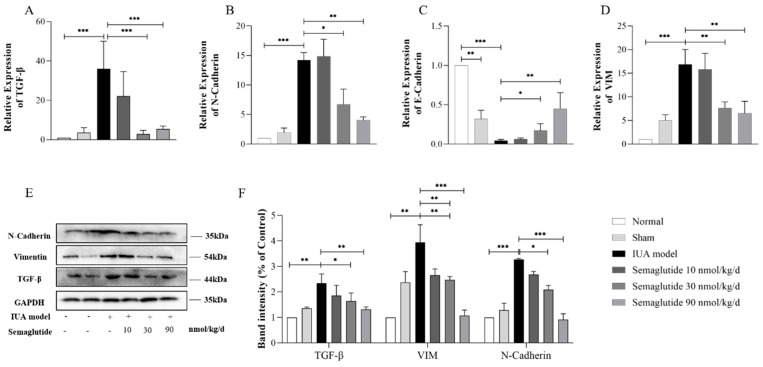
RT-qPCR analysis of the relative mRNA expression levels of TGF-β (**A**), N-Cadherin (**B**), E-Cadherin (**C**), and vimentin (**D**) in the uteri of mice in each group. Western blot analysis of the protein expression level of TGF-β, vimentin, and TGF-β in the uteri of mice in each group (**E**,**F**). Data are expressed as mean ± SD. * *p* < 0.05, ** *p* < 0.01, and *** *p* < 0.001.

The effect of Semaglutide on epithelial–mesenchymal transition in the uterine tissue of IUA mice is consistent with the cellular experiments. As shown in Figure 8, the expressions of TGF-β (*p* < 0.0001), VIM (*p* < 0.0001), and N-Cad (*p* < 0.0001) were increased and the expression of E-Cad (*p* < 0.0001) was decreased in the uterine tissues of IUA mice, but all changes were reversed after Semaglutide treatment.

## 3. Discussion

In this experiment, we used TGF-β1 to induce HEECs to establish an in vitro IUA model. Moreover, mechanical injury combined with lipopolysaccharide stimulation was used to establish a mouse IUA model. There is currently no consensus on evaluation criteria for in vitro IUA models, and they are mostly based on the expression of fibrosis markers [17]. H&E staining and Masson staining are usually used to evaluate the mouse IUA model, mainly observing the morphological changes, gland number, and collagen fiber deposition of the endometrium.

ACTA2 is a sign of cells transdifferentiating into myofibroblasts. The transformed cells can synthesize a large amount of collagen, giving the cells strong contractility and thus promoting the occurrence of fibrosis. COL1A1 is a type of collagen that plays an important role in scar formation. FN is a macromolecular extracellular membrane protein that exists on the surface of various animal cells. It is also the main non-collagenous glycoprotein in the extracellular matrix and basement membrane. It can mediate cell adhesion, promote cell migration, and promote fibroblast proliferation. In this experiment, the protein and mRNA expression of ACTA2 and the mRNA expression of FN and COL1A1 in the TGF-β1-induced HEEC model group were significantly increased, which indicated that the IUA cell model was successfully established. Through H&E staining and Masson staining, we can see that, compared with normal mice, IUA mice have morphological destruction and continuity interruption of the endometrium, a significant reduction in the number of glands, and a significant increase in collagen fiber deposition. These show that animal models have also been successfully established in this experiment. At the same time, we also detected changes in ACTA2, COL1A1, and FN in the uterine tissue of IUA mice, which were consistent with the changes in the cell model, which may indicate that the two IUA models in this experiment have certain similarities. After Semaglutide intervention, the expressions of ACTA2, COL1A1, and FN were significantly reduced in the IUA cell model, while in the mouse model, improvement in endometrial morphology, a significant increase in the number of glands, a decrease in collagen deposition, and a decrease in expression were observed of ACTA2, COLIAI, and FN. These show that Semaglutide can effectively improve intrauterine adhesions.

Inflammation is a key part of the IUA formation process. Although inflammation alone cannot directly lead to the occurrence of IUA, inflammation plays an important role in the occurrence and recurrence of IUA. Studies have shown that the NF-κB signaling pathway is an important way for stem cells to treat IUA [18,19]. Activating NF-κB can regulate the ratio of Th17/Treg cells, and Th17 can secrete IL-17, which can induce the activation of NF-κB and TNF-α receptor-related factors, resulting in an increased transcription of genes encoding IL-6. At the same time, IL-17 can combine with TNF-α to exert a synergistic effect on the above process by increasing the stability of mRNA and promoting the overexpression of TNF-α receptors. In addition, studies showed that the reduced expression of TNF-α and IL-6 is beneficial for promoting endometrial regeneration after injury in the IUA rat model [20,21]. In this experiment, we also detected TNF-α, IL-6, and NF-κB, which are closely related to inflammation and fibrosis, in two models. It can be seen that the expression levels of pro-inflammatory factors TNF-α, IL-6, and NF-κB, which are important in the inflammatory signaling pathway, are higher in cells and mice in the model group than in the normal group. After Semaglutide intervention, all the above indicators were significantly reduced, indicating that Semaglutide can reduce the inflammatory response in the IUA models.

In addition, reactive oxidative stress (ROS) processes are strongly associated with fibrosis. Under pathological conditions, NOX2 can produce higher concentrations of ROS through specific signaling pathways, thereby promoting pathological characteristics of the body, such as fibrosis, endothelial dysfunction, and heart failure [22,23]. A study on uterine fibrosis in female mice published in 2022 found that polystyrene microplastics activated the TLR4/NOX2 signaling axis, leading to oxidative stress, causing the activation of the Notch and TGF-β signaling pathways, and ultimately leading to uterine fibrosis [24]. We detected that the expression level of NOX2 increased significantly in TGF-β1-induced HEEC and animal models, which means that the oxidative stress process is closely related to IUA. However, after Semaglutide intervention, we only detected a decrease in NOX2 mRNA expression in the cell model and not in the IUA mice uterine. Therefore, whether the improvement of intrauterine adhesions by Semaglutide is related to the inhibition of ROS remains to be verified by further experiments.

We speculated that the inhibition of inflammation and fibrosis in IUAs by Semaglutide may be related to the inhibition of epithelial–mesenchymal transition, so we detected E-Cadherin, N-Cadherin, and vimentin, the key factors of epithelial–mesenchymal transition, in the two models. In this experiment, we can see that the expression levels of two mesenchymal cell markers, VIM and N-Cad, in the HEEC model were significantly higher than those in the control group, and the expression of the epithelial cell marker E-Cad was significantly lower than that in the control group. After the Semaglutide intervention, these indicators were closer to the normal group. 

Myofibroblasts are a differentiated form of fibroblasts and are a cell type used for matrix preservation, contributing to protein synthesis and fibrosis formation in the ECM. Epithelial cells can transform into mesenchymal fibroblasts through EMT under the influence of inflammation, trauma, and other factors. These cells also participate in tissue fibrosis repair. TGF-β expression is not only a necessary condition for the activation of interstitial fibroblasts but is also the most important factor in inducing EMT. At the same time, it can inhibit cell apoptosis, promote excessive secretion and deposition of ECM in tissues, and integrate with multiple signaling pathways to form a complex network, inducing the occurrence of fibrosis from many aspects [25]. We then detected TGF-βR and Snail1 in the cell model and TGF-β in the mouse model, and the results showed that their expression levels increased significantly in the model group and decreased in the administration group, indicating that Semaglutide inhibits the occurrence of EMT by affecting the expression of TGF-β, TGF-β receptors, and Snail1. However, more research is needed to prove the detailed mechanism and signaling cascade.

In summary, the novel GLP-1RA Semaglutide can improve fibrosis and inflammation in intrauterine adhesions, which may be related to its inhibition of epithelial–mesenchymal transition in the IUA models. This means it may be a promising drug for the treatment of intrauterine adhesions. More research is needed to explore the signaling cascade mechanism and targets of Semaglutide in improving intrauterine adhesions.

## 4. Methods

### 4.1. Chemicals

Semaglutide (98% purity) was purchased from GL Biochem (Shanghai, China). 

### 4.2. Animals and Animal Model Establishment

A total of 36 female C57BL/6J mice weighing 16–18 g were purchased from the Lanzhou Veterinary Research Institute of the Chinese Academy of Agricultural Sciences. The mice were housed in the Lanzhou University Animal Experiment Center at a temperature of 21 ± 3 °C and a 12 h light/12 h dark light cycle. The animals were allowed to eat and drink freely. All animal management and experimental procedures were performed in accordance with the Guidelines for the Care and Use of Laboratory Animals.

The animal model was induced as previously described [26]. Briefly, the IUA mouse model was dually induced by scraping the uterine cavity and burying surgical sutures with lipopolysaccharide (LPS) into the uterine cavity. Moreover, 48 h before surgery, the surgical sutures will be soaked in 0.6% LPS to fully absorb them. Thirty-six hours before surgery, each mouse was injected with 20 IU of pregnant mare serum gonadotropin to synchronize the estrous cycle. The mice were anesthetized by intraperitoneal injection of 100 uL 0.6% (*w*/*v*) sodium pentobarbital, and the uterus was exposed through an incision in the lower abdomen. A transverse incision of about 1 mm is made in the lower third of the uterus, and the uterine cavity is cured with a special curette. We stopped scraping when the uterine cavity was no longer smooth and when the uterine wall became rough and congested. Then, the surgical suture soaked in LPS was inserted into the uterine cavity through the uterine incision, passed through the abdominal wall head and tail, and knotted on the skin surface so that it could be removed after 48 h. Finally, each mouse was intraperitoneally injected with 100 ul of penicillin–streptomycin to prevent infection, and then the muscle layer and skin layer were sutured, respectively.

The 36 mice were evenly divided into six groups: a normal control group (normal, n = 6), a sham operation group (sham, n = 6), an IUA + double-distilled H_2_O (H_2_O) group (model, n = 6), an IUA + 10 nmol/kg Semaglutide group (n = 6), an IUA + 30 nmol/kg Semaglutide group (n = 6), and an IUA + 90 nmol/kg Semaglutide group (n = 6). Semaglutide was subcutaneously injected into the mice once a day for two weeks, while the control and sham-operated mice received equal amounts of sterile ddH2O. After 14 days of treatment, each mouse was sacrificed with an overdose of 4% chloral hydrate, and their uteri and blood were harvested.

### 4.3. Cell Culture and In Vitro Model Induction

HEECs were purchased from Procell Life Science & Technology Co., Ltd. (Wuhan, China) and cultured in DME/F12 supplemented with 10% FBS and 1% penicillin/streptomycin. The cells were maintained at 37 °C in a humidified atmosphere with 5% CO_2_. 

An in vitro cell model was induced by 10 ng/mL TGF-β1. After co-culturing with TGF-β1 with or without different concentrations of Semaglutide for 48 h, cells were collected for RT-qPCR and Western blotting analyses.

### 4.4. Cell Viability Assays

After being seeded in 96-well plates at a density of 5000 cells/well (200 μL/well) for 12 h, the cells were replaced with serum-free medium and cultured for 12 h. Then, the cells were treated with different concentrations of Semaglutide (2.5 μM, 5 μM, 10 μM, and 20 μM) with or without TGF-β1. After 48 h, CCK-8 (Cell Counting Kit-8, Coolaber, Beijing, China) solution (10 μL) was added to each well and incubated for another 2 h, respectively. The absorbance was measured at 450 nm using a microplate reader (Infinite M200PRO, TECAN, Grödig, Austria).

### 4.5. Histomorphological Evaluation

The harvested uteri were fixed with 4% paraformaldehyde for 48 h, embedded in paraffin, and cut into sections that were 4 μm in thickness. Hematoxylin and eosin (H&E) and Masson staining were performed according to the reagent instructions. After drying, the sections were mounted with neutral resin. All sections were photographed using an Olympus BX73 microscope (Olympus Optical Co., Tokyo, Japan) equipped with an OCULAR camera.

Two slices of the uterus of each animal were stained, and all areas were counted for statistics. Glands in the submucosa and basal layer of the uterine slices were counted at 200x magnification, and their average numbers were calculated. The extent of endometrial fibrosis was assessed by quantifying the area occupied by blue-stained collagen fibers in each Masson-stained slice. The blue-stained area of collagen was quantified with Image J 8.0 software.

### 4.6. ELISA Assay

Blood from the mice was collected through a cardiac puncture. After centrifugation at 3000 r for 20 min at 4 °C, the serum was collected and stored at −20 °C. The detection of IL-6 and TNF-α was performed using their corresponding ELISA kits (Jianglin, Shanghai, China).

### 4.7. RNA Extraction and RT-qPCR

Total RNA was extracted from harvested cells and animal uteri, respectively, according to the total RNA extraction reagent instructions. Using the RevertAid First Strand cDNA Synthesis Kit (Yeasen, Shanghai, China), RNA was reverse-transcribed into cDNA in one step. Target gene expression was evaluated using a QuantStodio TM 3 Real-Time PCR Instrument (Thermo Fisher Scientific, Waltham, MA, USA). All primers used in this experiment are listed in Table 1. The PCR cycling parameters were as follows: 95 °C for 10 s, followed by 40 cycles of 95 °C for 30 s, 55 °C for 30 s, and 72 °C for 30 s. Relative mRNA expression levels were determined using the comparative Ct (ΔΔCt) method.

### 4.8. Western Blotting

Total protein samples were isolated from harvested cells and animal uteri using RIPA lysis buffer, following reagent instructions. Protein concentration was detected using the BCA protein assay kit (Transgen Biotech, Beijing, China). Samples containing 20 μg of protein were separated on a 10% SDS-PAGE gel and then transferred to a polyvinylidene difluoride membrane (PVDF, Merck Millipore, Darmstadt, Germany). After the PVDF membrane was blocked with TBST containing 5% skimmed milk powder, it was incubated with a specific ratio of primary antibodies overnight at 4 °C. After washing with TBST, the membrane was incubated with horseradish peroxidase-conjugated anti-rabbit IgG or anti-mouse IgG secondary antibodies. The antibody ratios and information used in this experiment are shown in Table 2. The generated signal was acquired using a protein imaging system (Glarity Western ECL Substrate, Bio-Rad, Hercules, CA, USA). Protein expression levels were quantified using Image J 8.0 software and normalized to loading control β-actin.

### 4.9. Statistical Analysis

All data are presented as mean ± standard deviations (SD), and all data were analyzed using GraphPad Prism version 9. When the data were normally distributed, differences between the two groups were analyzed using the Student’s *t* test. Differences between three or more groups were analyzed using one-way analysis of variance (ANOVA) and the Student–Newman–Keuls multiple comparison test. Differences were considered statistically significant at *p* < 0.05.

## Figures and Tables

**Table 1:** Primers used for quantitative reverse transcription polymerase chain reaction.

Gene	Forward Primer (5′-3′)	Reverse Primer (5′-3′)
*Acta2 -Homo*	CGGGACATCAAGGAGAAACTG	CCATCAGGCAACTCGTAACTCT
*β-actin -Homo*	TGGCACCCAGCACAATGAA	CTAAGTCATAGTCCGCCTAGAAGCA
*CDH2 -Homo*	CTCGGTGGATATGGTCCTTCTCTTC	CTGGTACACAATACAGAGGCAAAGC
*COL1A1 -Homo*	GATGCCATCAAAGTCTTCTGCA	GGAATCCATCGGTCATGCTCT
*FN -Homo*	ATCAAGTATGAGAAGCCTGGGTC	AGTTGGGGAAGCTCGTCTGT
*IL-6 -Homo*	CACTGGTCTTTTGGAGTTTGAGG	CTGGCATTTGTGGTTGGGT
*NF-κB -Homo*	CCGTTGGGAATGGTGAGGTC	CGCGTAGTCGAAAAGGGCAT
*NOX2 -Homo*	AGTGCGTGCTGCTCAACAAGA	GACTCGGGCATTCACACACCATT
*SNIAL1 -Homo*	GACCCCAATCGGAAGCCTAA	AGGGCTGCTGGAAGGTAAAC
*TGF-βR -Homo*	CAGTCATTAGAGGTGGAAGGGAG	TGAAGTCAAAGCAGGGGCAT
*TNF-α -Homo*	CCTGCCCCAATCCCTTTA	GTGGTTGCCAGCACTTCACT
*VIM -Homo*	GGACCAGCTAACCAACGACA	AAGGTCAAGACGTGCCAGAG
*Acta2 -mus*	TTCGTGACTACTGCCGAGC	GTCAGGCAGTTCGTAGCTCT
*β-actin -mus*	CTGAGAGGGAAATCGTGCGT	TGTTGGCATAGAGGTCTTTACGG
*CDH1 -mus*	TGCTGCTCCTACTGTTTCTACGG	CCACATCATTTCGAGTCACTTCC
*CDH2 -mus*	CGGCATACAGAATCAGTGGTGGAG	GCAGCAACAGTGAGGACAAACATC
*COL1A1 -mus*	GCTCCCTTGGACATTGGTG	ATTGAGTTTGGGTTGTTCGTCT
*FN1 -mus*	GCACAAGGTTCGGGAAGAGG	GCGTAATGGGAAACCGTGTAA
*NF-κB -mus*	TGCCAAAGAAGGACACGACA	TGAGCATTGACTTCTGCCCC
*NOX2 -mus*	TGCCCCAAGGTATCCAAGTT	CATTGAATAGCCCCTCCGTC
*TGF-β -mus*	GCCTGAGTGGCTGTCTTTTG	GCCCTGTATTCCGTCTCCTT
*VIM -mus*	TCCAGAGAGAGGAAGCCGAA	TTCAAGGTCAAGACGTGCCA

**Table 2:** Primary and secondary antibodies used for Western blotting.

Antibody	Host	Dilution	Company	Catalog No.
Anti-TGF-β	Rabbit	1:1000	Cell Signaling Technology	#3709
Anti-N-Cadherin	Rabbit	1:2000	Cell Signaling Technology	#8828
Anti-E-Cadherin	Mouse	1:1000	Cell Signaling Technology	#14472
Anti-Vimentin	Mouse	1:1000	Cell Signaling Technology	#5741
Anti-Acta2	Mouse	1:1000	Cell Signaling Technology	#11948
Anti-GAPDH mouse monoclonal antibody	Mouse	1:5000	TRANS	HC301-01
Goat-anti-mouse IgG	Goat	1:10000	OriGene	ZB-2305
Goat-anti-rabbit IgG	Goat	1:10000	OriGene	ZB-2301

## Data Availability

The authors declare that all the data supporting the findings of this study are contained within the paper.

## References

[1] Shen M., Duan H., Chang Y., Lin Q. (2022). Prevalence and risk factors of intrauterine adhesions in women with a septate uterus: A retrospective cohort study. Reprod. Biomed. Online.

[2] Hooker A.B., Lemmers M., Thurkow A.L., Heymans M.W., Opmeer B.C., Brölmann H.A., Mol B.W., Huirne J.A. (2014). Systematic review and meta-analysis of intrauterine adhesions after miscarriage: Prevalence, risk factors and long-term reproductive outcome. Hum. Reprod. Update.

[3] Xu W., Zhang Y., Yang Y., Zhang S., Lin X. (2018). Effect of early second-look hysteroscopy on reproductive outcomes after hysteroscopic adhesiolysis in patients with intrauterine adhesion, a retrospective study in China. Int. J. Surg..

[4] Yu D., Wong Y.M., Cheong Y., Xia E., Li T.C. (2008). Asherman syndrome—One century later. Fertil. Steril..

[5] March C.M. (2011). Management of Asherman’s syndrome. Reprod. Biomed. Online.

[6] Sutton C. (2006). Hysteroscopic surgery. Best Pract. Res. Clin. Obstet. Gynaecol..

[7] Lee W.L., Liu C.H., Cheng M., Chang W.H., Liu W.M., Wang P.H. (2021). Focus on the Primary Prevention of Intrauterine Adhesions: Current Concept and Vision. Int. J. Mol. Sci..

[8] Cao J., Liu D., Zhao S., Yuan L., Huang Y., Ma J., Yang Z., Shi B., Wang L., Wei J. (2020). Estrogen attenuates TGF-β1-induced EMT in intrauterine adhesion by activating Wnt/β-catenin signaling pathway. Braz. J. Med. Biol. Res.=Rev. Bras. Pesqui. Medicas Biol..

[9] Liu L., Huang X., Xia E., Zhang X., Li T.C., Liu Y. (2019). A cohort study comparing 4 mg and 10 mg daily doses of postoperative oestradiol therapy to prevent adhesion reformation after hysteroscopic adhesiolysis. Hum. Fertil..

[10] Christou G.A., Katsiki N., Blundell J., Fruhbeck G., Kiortsis D.N. (2019). Semaglutide as a promising antiobesity drug. Obes. Rev. Off. J. Int. Assoc. Study Obes..

[11] Ussher J.R., Drucker D.J. (2014). Cardiovascular actions of incretin-based therapies. Circ. Res..

[12] Noyan-Ashraf M.H., Shikatani E.A., Schuiki I., Mukovozov I., Wu J., Li R.K., Volchuk A., Robinson L.A., Billia F., Drucker D.J. (2013). A glucagon-like peptide-1 analog reverses the molecular pathology and cardiac dysfunction of a mouse model of obesity. Circulation.

[13] Li Y.K., Ma D.X., Wang Z.M., Hu X.F., Li S.L., Tian H.Z., Wang M.J., Shu Y.W., Yang J. (2018). The glucagon-like peptide-1 (GLP-1) analog liraglutide attenuates renal fibrosis. Pharmacol. Res..

[14] Oztay F., Sancar-Bas S., Gezginci-Oktayoglu S., Ercin M., Bolkent S. (2018). Exendin-4 partly ameliorates-hyperglycemia-mediated tissue damage in lungs of streptozotocin-induced diabetic mice. Peptides.

[15] Patel V., Joharapurkar A., Kshirsagar S., Sutariya B., Patel M., Patel H., Pandey D., Patel D., Ranvir R., Kadam S. (2018). Coagonist of GLP-1 and Glucagon Receptor Ameliorates Development of Non-Alcoholic Fatty Liver Disease. Cardiovasc. Hematol. Agents Med. Chem..

[16] Fang S., Cai Y., Li P., Wu C., Zou S., Zhang Y., Lin X., Guan M. (2019). Exendin-4 alleviates oxidative stress and liver fibrosis by activating Nrf2/HO-1 in streptozotocin-induced diabetic mice. Nan Fang Yi Ke Da Xue Xue Bao=J. South. Med. Univ..

[17] Thiery J.P., Sleeman J.P. (2006). Complex networks orchestrate epithelial-mesenchymal transitions. Nat. Rev. Mol. Cell Biol..

[18] Hua Q., Zhang Y., Li H., Li H., Jin R., Li L., Xiang Y., Tian M., Wang J., Sun L. (2022). Human umbilical cord blood-derived MSCs trans-differentiate into endometrial cells and regulate Th17/Treg balance through NF-κB signaling in rabbit intrauterine adhesions endometrium. Stem Cell Res. Ther..

[19] Bettelli E., Carrier Y., Gao W., Korn T., Strom T.B., Oukka M., Weiner H.L., Kuchroo V.K. (2006). Reciprocal developmental pathways for the generation of pathogenic effector TH17 and regulatory T cells. Nature.

[20] Gan L., Duan H., Xu Q., Tang Y.Q., Li J.J., Sun F.Q., Wang S. (2017). Human amniotic mesenchymal stromal cell transplantation improves endometrial regeneration in rodent models of intrauterine adhesions. Cytotherapy.

[21] Zhang L., Li Y., Guan C.Y., Tian S., Lv X.D., Li J.H., Ma X., Xia H.F. (2018). Therapeutic effect of human umbilical cord-derived mesenchymal stem cells on injured rat endometrium during its chronic phase. Stem Cell Res. Ther..

[22] Lassègue B., San Martín A., Griendling K.K. (2012). Biochemistry, physiology, and pathophysiology of NADPH oxidases in the cardiovascular system. Circ. Res..

[23] Panera N., Braghini M.R., Crudele A., Smeriglio A., Bianchi M., Condorelli A.G., Nobili R., Conti L.A., De Stefanis C., Lioci G. (2022). Combination Treatment with Hydroxytyrosol and Vitamin E Improves NAFLD-Related Fibrosis. Nutrients.

[24] Wu H., Xu T., Chen T., Liu J., Xu S. (2022). Oxidative stress mediated by the TLR4/NOX2 signalling axis is involved in polystyrene microplastic-induced uterine fibrosis in mice. Sci. Total Environ..

[25] Zhu H.Y., Ge T.X., Pan Y.B., Zhang S.Y. (2017). Advanced Role of Hippo Signaling in Endometrial Fibrosis: Implications for Intrauterine Adhesion. Chin. Med. J..

[26] Ma X.L., Ding Y., Wu L.M., Wang Y.X., Yao Y., Wang Y.X., Zhang Y.G., Niu J.Q., He X.X., Wang Y.Q. (2021). The glucagon-like peptide-1 (GLP-1) analog exenatide ameliorates intrauterine adhesions in mice. Peptides.

